# Antibodies against apoB100 peptide 210 inhibit atherosclerosis in apoE^-/-^ mice

**DOI:** 10.1038/s41598-021-88430-1

**Published:** 2021-04-27

**Authors:** Pontus Dunér, Ingrid Yao Mattisson, Per Fogelstrand, Lars Glise, Stacey Ruiz, Christopher Farina, Jan Borén, Jan Nilsson, Eva Bengtsson

**Affiliations:** 1 Department of Clinical Sciences Malmö, Clinical Research Centre, Lund University, Jan Waldenströms street 35, 20502 Malmö, Sweden; 2 Department of Molecular and Clinical Medicine, Wallenberg Laboratory, Institute of Medicine, The Sahlgrenska Academy, University of Gothenburg and Sahlgrenska University Hospital, Göteborg, Sweden; 3 Abcentra LLC, Los Angeles, CA USA; 4 Present Address: Redoxis AB, Medicon Village, Lund, Sweden

**Keywords:** Cardiology, Diseases

## Abstract

Atherosclerotic plaques are characterized by an accumulation and subsequent oxidation of LDL, resulting in adaptive immune responses against formed or exposed neoepitopes of the LDL particle. Autoantibodies against native p210, the 3136–3155 amino acid sequence of the LDL protein apolipoprotein B-100 (apoB100) are common in humans and have been associated with less severe atherosclerosis and decreased risk for cardiovascular events in clinical studies. However, whether apoB100 native p210 autoantibodies play a functional role in atherosclerosis is not known. In the present study we immunized apoE^-/-^ mice with p210-PADRE peptide to induce an antibody response against native p210. We also injected mice with murine monoclonal IgG against native p210. Control groups were immunized with PADRE peptide alone or with control murine monoclonal IgG. Immunization with p210-PADRE induced an IgG1 antibody response against p210 that was associated with reduced atherosclerotic plaque formation in the aorta and reduced MDA-LDL content in the lesions. Treatment with monoclonal p210 IgG produced a similar reduction in atherosclerosis as immunization with p210-PADRE. Our findings support an atheroprotective role of antibodies against the apoB100 native p210 and suggest that vaccines that induce the expression of native p210 IgG represent a potential therapeutic strategy for lowering cardiovascular risk.

## Introduction

Cardiovascular disease, including myocardial infarction and stroke, is the leading cause of death globally^1^. The underlying cause is a build-up of atherosclerotic plaques in the arteries due to an accumulation of low-density lipoprotein (LDL) particles. These LDL particles become oxidized in the vascular wall resulting in activation of adaptive immune responses against neo-epitopes formed or exposed as result of the oxidation process^2–5^. Immunogenic epitopes on LDL exist in the form of oxidized phospholipid motifs and native and aldehyde-modified peptides of apolipoprotein B100 (apoB100), which is the protein component of LDL. Screening of a peptide library containing 302 peptides comprising the complete apoB100 sequence with human plasma, identified both native and malondialdehyde (MDA)-modified apoB100 peptide 210 (p210) as important epitopes recognized by autoantibodies^6^.

The association of autoantibodies against native or MDA-p210 with cardiovascular disease has subsequently been investigated in several large population and case–control cohorts. IgG autoantibodies against the native form of p210 have been associated with less severe atherosclerosis and a lower risk of cardiovascular events, whereas IgG against MDA-p210 often failed to show this association. High levels of IgG against native p210 were inversely associated with the degree of coronary atherosclerosis in 243 patients from the SCARF study^7^. The same study also showed that IgG autoantibodies against native p210, but not against MDA-p210, were lower in 387 post-myocardial infarction patients compared to 387 age-sex matched healthy controls^7^. Furthermore, baseline plasma levels of IgG against native p210 were inversely associated with carotid intima-media thickness in 3430 participants of the IMPROVE study^8^. Recently, it was shown that baseline levels of IgG against native p210, but not against MDA-p210, were lower in individuals who later suffered from myocardial infarction in the Malmö Diet and Cancer cohort including 5393 participants^9^. In addition, subjects with high levels of IgG against native p210 were less likely to have carotid plaques^9^. Accordingly, there is extensive clinical evidence for an association of IgG autoantibodies against native p210 and a reduced risk of cardiovascular disease. Whereas immunization of hypercholesterolemic mice with MDA-p210 has been shown to induce antigen-specific antibodies and reduce atherosclerotic plaque formation^10^, immunization studies using native p210 inhibit the development of atherosclerosis without induction of p210 autoantibodies^11–13^. Instead, the protective effect of native p210 immunizations was attributed to an increase in regulatory T cells^13^ or CD8 cells^11^. Thus, it remains an open question as to whether IgG against native p210 play a functional role in protecting against cardiovascular disease or whether, alternatively, low levels of these autoantibodies only serve as non-functional markers of future cardiovascular disease.

To test the hypothesis that autoantibodies against native p210 protect against atherosclerosis, we either immunized atherosclerotic apoE^-/-^ mice with apoB100 p210 conjugated to Pan-DR T cell epitopes (PADRE), a potent immunogenic amino acid sequence^14–16^, or treated them with a monoclonal antibody specifically targeted against apoB100 p210.

## Results

### Immunization with apoB100 p210 conjugated to PADRE induces peptide specific antibodies

**Figure 1:**
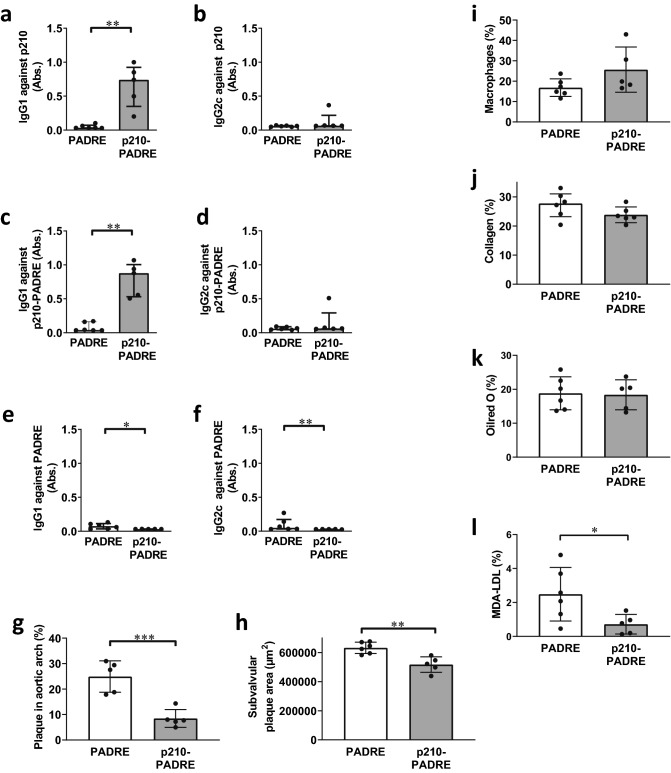
ApoE^-/-^ mice immunized with native p210-PADRE develop p210-specific IgG1 antibodies and less atherosclerosis. ApoE^-/-^ mice were immunized with native p210-PADRE or PADRE peptide. IgG1 (**a**, **c**, **e**) and IgG2c (**b**, **d**, **f**) antibodies against p210 (**a**, **b**), p210-PADRE (**c**, **d**), or PADRE (**e**, **f**) (plasma dilution 1:1000) from immunized mice were analyzed in ELISA. Atherosclerotic plaque formation was analyzed in the aortic arch (**g**) and in subvalvular plaques (**h**). Macrophage (**i**), collagen (**j**), lipid (**k**), and MDA-LDL (**l**) content were analyzed in subvalvular plaques. Data are depicted as individual mice, with bars indicating median (IQR) (**a**–**f**) or mean ± SD (**g**–**k**). *p < 0.05, **p < 0.01, ***p < 0.001; Mann–Whitney test (**a**–**f**) or Unpaired t-test (**g**–**l**).

Previous immunizations studies in mice using native p210 coupled to albumin did not result in induction of an antibody response against p210^12, 13^. In this study, we therefore tested whether coupling of native p210 to the immunogenic sequence PADRE would result in induction of a p210 antibody response. In accordance with this notion we found that immunization of *Apoe*^*-/-*^ mice with p210-PADRE using Alum as adjuvant induced a strong antibody response against p210, whereas mice immunized with PADRE alone did not exhibit p210 antibodies (Fig. 1a–d, Supplementary Fig. 1). Importantly, these autoantibodies were specific for p210 and did not react with PADRE alone (Fig. 1e–f). The induced antibodies were of IgG1 type, corresponding to a Th2 response (Fig. 1a,c). No IgM antibodies against p210 or PADRE were detected (Supplementary Fig. 2). To determine if the induction of p210-antibodies was accompanied by a cytokine response, we analyzed inflammatory, Th1-, Th2-, Treg-, and Th17-associated cytokines in plasma and from splenocytes of immunized mice (Supplementary Figs. 3–4). Immunization did not affect the Th2-associated cytokines IL-4 and IL-5, Treg-associated IL-10, Th17-associated IL-17, or the inflammatory cytokine TNFα. However, the Th2 cytokine IL-13 and the inflammatory cytokine IL-6 were decreased in plasma from P210-PADRE immunized mice. Splenocytes from P210-PADRE immunized mice displayed an increase in the Th2 cytokine IL-13 and in the Th1 cytokine IFNγ, whereas the expression of monocyte chemoattractant protein-1 (MCP-1) was decreased as compared to splenocytes from PADRE-immunized mice.

### Immunization with apoB100 p210 inhibits atherosclerosis and lowers MDA-LDL content in the lesions

Next, we tested whether immunization with p210-PADRE affected plasma lipid levels. However, neither cholesterol nor triglyceride levels differed between p210-PADRE and PADRE-immunized mice (Supplementary Table 1). Furthermore, plasma levels of oxidized LDL were not affected by immunization (Supplementary Table 1). P210-PADRE-immunized mice did not differ in body weight compared to PADRE-immunized control mice (Supplementary Table 1).

Mice immunized with p210-PADRE displayed a 66% decrease in plaque area in the aortic arch and an 18% decrease in subvalvular plaques compared to mice immunized with PADRE alone (Fig. 1g,h, Supplementary Fig. 5). In addition to smaller plaques, p210-PADRE immunization resulted in a trend to decreased lipid content (178,000 ± 51,000 vs 228,000 ± 26,000 µm^2^, *p* = 0.06) in subvalvular plaques. Immunization with p210-PADRE did not affect the relative content of macrophages, collagen, or lipids of subvalvular plaques (Fig. 1i–k, Supplementary Fig. 5). However, aldehyde-modified (MDA)-LDL content in the lesions was significantly decreased in p210-PADRE-immunized mice compared to control mice (Fig. 1l, Supplementary Fig. 5).

### Antibodies against apoB100 p210 protect against atherosclerosis

**Figure 2:**
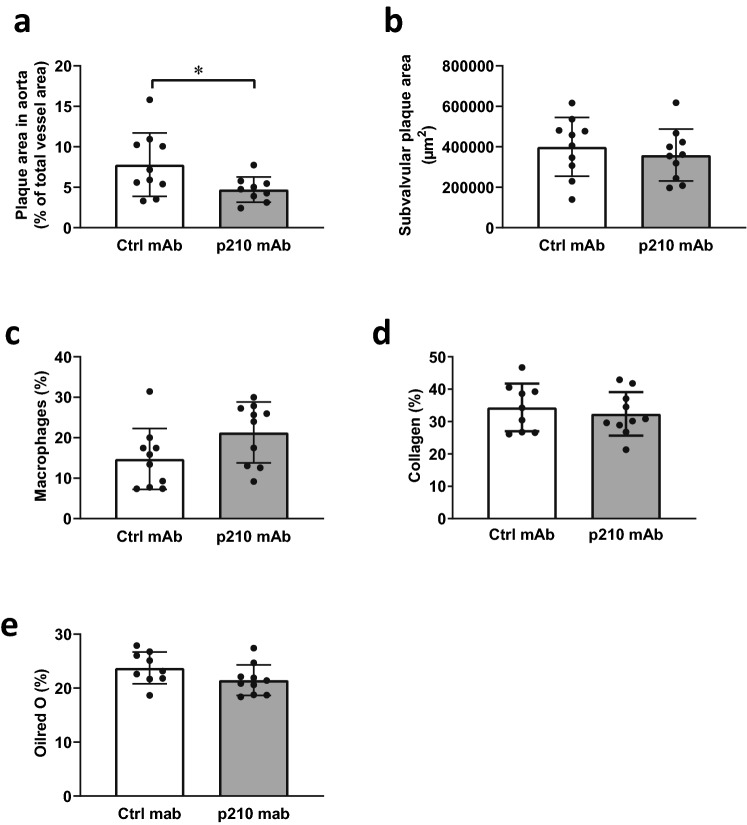
Mice immunized with monoclonal IgG against native p210 develop less atherosclerosis. ApoE^-/-^ mice were immunized with monoclonal IgG against native p210 or control IgG, and atherosclerotic plaque formation were analyzed in the aortic arch (**a**) and in subvalvular plaques (**b**). Macrophage (**c**), collagen (**d**), and lipid (**e**) content were measured in subvalvular plaques. Data are depicted as individual mice, with bars indicating mean ± SD (**a**–**e**). *p < 0.05; Unpaired t-test.

Finally, to investigate if the atheroprotective effect was mediated by p210-induced antibodies, we passively immunized *Apoe*^*-/-*^ mice with a monoclonal antibody directed against native p210 (p210 mAb), while control mice received murine monoclonal IgG (ctrl mAb). Treatment with p210 mAb did not affect plasma cholesterol, triglyceride, oxidized LDL levels or body weight compared to control mice (Supplementary Table 2). Furthermore, inflammatory, Th1-, Th2-, Treg-, or Th17-associated cytokine secretion from splenocyte cultures did not differ between p210 mAb-treated mice and control mice (Supplementary Fig. 6). However, mice injected with p210 mAb exhibited a 39% decrease in aortic plaque area compared to control mice, whereas subvalvular plaque areas were not affected (Fig. 2a,b, Supplementary Fig. 7). The plaque relative content of macrophages, collagen, or lipids did not differ significantly between the groups (Fig. 2c–e, Supplementary Fig. 7). MDA-LDL staining was not observed in any of the mice in the apoB100 p210 mAb-group. In the ctrl mAb group two mice displayed minor MDA-LDL staining, whereas the rest of the group was negative.

### Antibodies against apoB100 p210 do not inhibit LDL binding to the vessel wall

**Figure 3:**
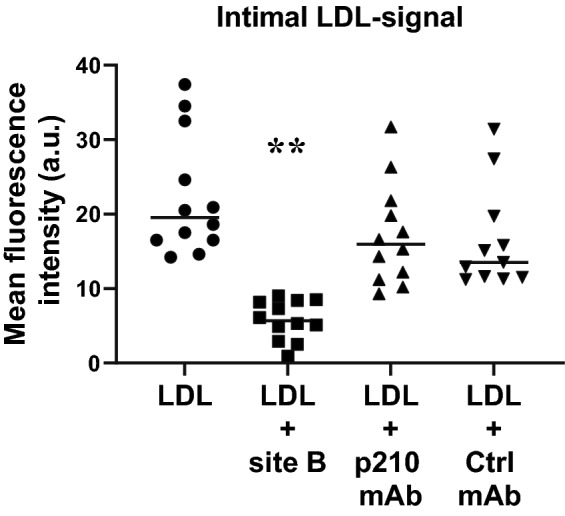
Native p210 antibodies do not affect electrostatic LDL binding to the vessel wall. Cryosections from mouse carotid arteries with intimal hyperplasia were incubated with fluorophore-labelled LDL in the presence of native p210 mAb or ctrl Ab. Positively charged peptide (site B peptide) was used as a positive control to block electrostatic binding of LDL to the vessel wall.**p < 0.01 compared to the ctrl mAb and *p* < 0.001 compared to LDL and p210 mAb.

LDL retention in the vessel wall is mediated via binding of positively charged arginine and lysine residues in site B of apoB100 to negatively charged extracellular matrix proteoglycans^17^, resulting in atherosclerotic plaque development^18^. Since the amino acid sequence of p210 contain six lysine residues, we hypothesized that antibodies against native p210 would block LDL interaction to matrix components in the vessel wall, and that the observed decreased MDA-LDL content in the native p210 immunized mice may be secondary to an effect of inhibited LDL retention. To test this, we performed an ex vivo LDL binding experiment where intimal hyperplasia was induced in the right common carotid artery of mice, and sections from the artery were incubated with fluorescently labelled LDL in the presence of p210 mAb IgG or control IgG. However, p210 mAb IgG did not affect LDL binding to the artery (Fig. 3), arguing that the atheroprotective effect of the antibody is not mediated via reduced LDL retention in the artery wall.

### p210 mAb IgG binds p210, MDA-apoB100 and MDA-LDL

**Figure 4:**
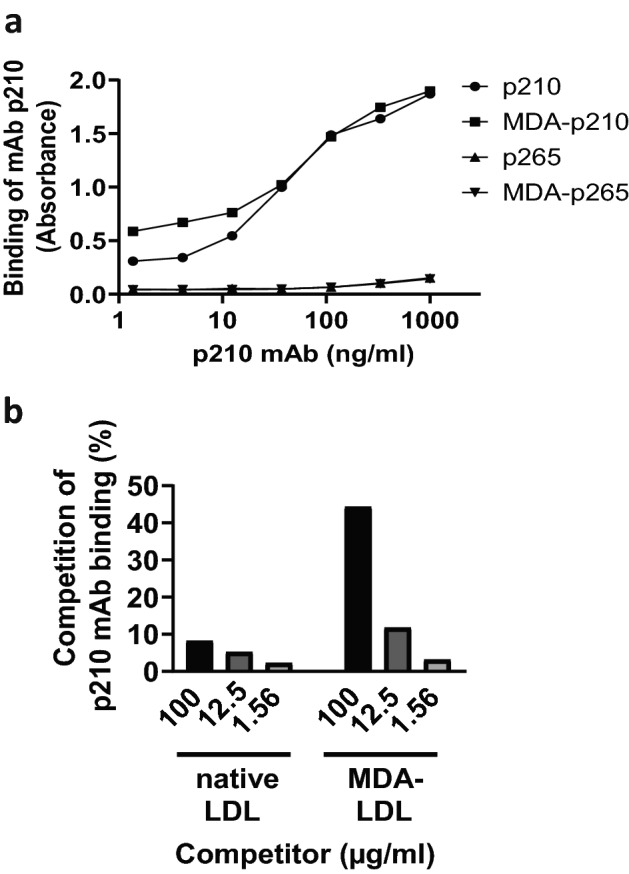
p210 mAb binds p210 and MDA-modified LDL. The specificity of p210 mAb was analyzed in ELISA. (**a**) Microtiter wells were coated with native or MDA-modified forms of p210 or apoB100 peptide p265, and binding of p210 mAb was analyzed. (**b**) Microtiter wells were coated with native p210, and binding of p210 mAb was competitively inhibited by addition of native or MDA-modified LDL.

Finally, we tested the binding of p210 mAb to native or modified forms of p210, apoB100 and LDL. The specificity of p210 mAb to p210 and not to other apoB100 peptides was confirmed in ELISA, where p210 mAb bound both native and MDA-p210, but not native or MDA-p265 (Fig. 4a). The binding of p210 mAb to native p210 was competitively inhibited by addition of MDA-LDL, but not by native LDL (Fig. 4b). Furthermore, p210 mAb bound MDA-apoB100 (K_D_ = 2.86 × 10^−10^) using Octet binding assay, whereas no binding to native apoB100 was detected (Table 1).

**Table 1:** Analysis of p210 mAb with Octet binding assay.

Analyte	On Rate (k_a_)	Off Rate (k_d_)	Equilibrium (K_D_)
ApoB100	NA	NA	NA
MDA-apoB100	1.85 × 10^−6^	5.28 × 10^−4^	2.86 × 10^−10^

*NA* not analyzed, no binding detected.

## Discussion

Autoantibodies against the native unmodified form of apoB100 p210 have been associated with less severe atherosclerosis and lower risk for development of cardiovascular events in several large cohorts^7–9, 19^. However, whether these autoantibodies have a protective function in atherosclerosis is not known. In the present study we show that immunization of mice with native p210 coupled to PADRE peptide results in autoantibodies against apoB100 p210 and protects against atherosclerotic lesion development, and that treatment with native p210 IgG mAb has a similar effect on lesions in the aorta.

Interestingly, in a recent study by Zeng et al., immunization with MDA-modified p210 using Freund’s complete adjuvant induced antigen specific antibodies and inhibited atherosclerotic plaque development^10^. Furthermore, passive immunization with polyclonal IgG against MDA-p210 had a similar effect^10^. Whereas IgG against native p210 in plasma are inversely associated with cardiovascular disease in human cohort studies, IgG against MDA-p210 in many cases fail to display this association^7, 9^. Thus, it is intriguing that immunization of mice with either native p210 in the current study or with MDA-p210 by Zeng *et al*^10^ result in decreased atherosclerosis. It has been argued that autoantibodies against native p210 recognize mildly oxidized LDL, whereas autoantibodies against MDA-p210 recognize more heavily oxidized LDL, which may not be as relevant in the actual human disease, explaining the findings in human cohorts. Antibodies recognize in general five to six amino acids, and the p210 peptide used in the MDA-p210 immunization study by Zeng et al. was 20 amino acid long^10^. Furthermore, MDA modifies mainly lysine, arginine, and histidine residues, thus it is likely that antibodies against both MDA- and non-modified amino acid epitopes could be induced upon immunization with MDA-p210. Active immunization using MDA-p210 and passive immunization with polyclonal antibodies raised against MDA-p210^10^ may then include antibodies against MDA-modified as well as native p210 epitopes, which both may affect atherosclerotic disease. Interestingly, another study using recombinant human antibodies against MDA-p210 (KTT-D6) specifically selected for MDA-modified peptides, did not result in reduced lesion formation in apoE^-/-^ mice^20^. The current study showing that both active and passive immunization with native p210 IgG results in reduced atherosclerotic plaque development in mice support the notion that autoantibodies against the p210 epitope are atheroprotective. Importantly, it also connects previous human cohort studies of native p210 IgG and cardiovascular disease with a functional role of these autoantibodies in vivo.

Previous in vitro studies revealed that antibodies against MDA-p210 reduce oxidized LDL accumulation in macrophages and promote cholesterol efflux^10^. Notably, in the present study, we show that immunization with native p210 reduces the actual MDA-LDL content in atherosclerotic plaques. In line with this finding, we discovered that mAb p210 binds MDA-apoB100 and MDA-LDL, but not native apoB100 or native LDL, suggesting that the p210 epitope is exposed upon MDA-modification of LDL. These data are in accordance with the proposal that autoantibodies against native p210 recognize mildly oxidized LDL, and suggests that the atheroprotective effect of native p210 antibodies is mediated by removal of MDA-LDL from the lesions, similar to the in vitro mechanism described for antibodies induced against MDA-p210. In p210 mAb treated mice, MDA-LDL staining was present in lesions from two control mice, but in none of the p210 mAb treated mice*.* The result that MDA-LDL is decreased in p210-PADRE immunized mice, but not present in the majority of subvalvular lesions of mAb p210 treated mice is intriguing. A possible explanation may be that subvalvular plaques from passively immunized mice were smaller, and therefore at an earlier stage with a lower MDA-LDL content.

Previous immunization studies in mice using native p210 and bovine serum albumin as a carrier resulted in reduced atherosclerosis, in the absence of a p210 antibody response or presence of only a minor p210 antibody response^12, 21^. Additional studies have shown that immunization with native p210-albumin results in the generation of regulatory T cells^13^ or CD8 T cells^22^, which reduced atherosclerosis. In the present study, we induced a robust antibody response against native p210 in immunized mice by coupling p210 to the immunogenic peptide PADRE^14, 16^, which is known to generate strong antibody responses in apoE^-/-^ mice^15^. The induced p210-specific antibodies were of Th2 type, which is in line with the use of Alum as adjuvant^23^. To investigate if the atheroprotective effect of immunization was due to a concomitant increase in Th2-associated cytokines, we analyzed the secretion of IL-4, IL-5 and IL-13 cytokines in plasma and from cultured splenocytes obtained from immunized mice. The Th2 associated cytokines IL-4 and IL-5 were not affected, while IL-13 secretion was decreased in plasma but increased in splenocytes from native p210-immunized mice compared to control mice. Since IL-13 has previously been shown to protect against atherosclerosis in mice^24^, it is possible that the observed decrease in atherosclerosis in the present study could partially be attributed to altered IL-13 levels. Furthermore, we also observed a decrease in systemic IL-6 levels in p210-PADRE immunized mice, which may have an effect lesion size. On the other hand, p210-PADRE immunized mice displayed an increase in the Th1-associated cytokine IFNγ. IFNγ^25–27^, its receptor^28^, or the Th1 differentiation transcription factor Tbet^29^ have consistently been proven to aggravate atherosclerosis in mice. Thus, the increase in IFNγ in the current study is likely to at least in part counteract the possible atheroprotective effects of other cytokines. Importantly, immunization with IgG p210 mAb inhibited atherosclerotic lesion development without affecting Th1, Th2, Treg, or inflammatory cytokines in splenocytes, suggesting that these antibodies have a protective effect that is independent of cytokines. However, active immunization using p210-PADRE had a more pronounced effect on atherosclerotic lesion size than passive immunization with p210 mAb. Thus, it is likely that the atheroprotection seen upon p210-PADRE immunization is partly due to additional mechanisms, such as alterations of systemic cytokines, apart from the induction of p210 specific antibodies.

The present study has some limitations, which need to be considered. Most importantly, results in mice should be interpreted with due caution when comparing with human data. For example, the earliest vascular change in human vessels is an intimal thickening, consisting of smooth muscle cells and extracellular matrix. This is then followed by an accumulation of lipids and macrophages^30^. In mice, lipids and macrophages accumulate first, whereas smooth muscle cell migration from the media to the intima occurs at a later stage. Furthermore, atherosclerosis development per se in mice does not result in myocardial infarctions as in humans, which makes it difficult to compare measurements of murine plaques with the risk for cardiovascular events in humans.

## Conclusion

This is the first study showing that autoantibodies against native p210 protect against atherosclerotic plaque development. The result is in accordance with previous human studies, showing an inverse association with apoB100 native p210 IgG and plaques in the coronary or carotid arteries, and indicates that the autoantibodies measured in human cohorts indeed play a functional role in atherosclerosis. They also suggest that vaccines that induce the expression of apoB100 native p210 IgG represent a possible approach for lowering cardiovascular risk.

## Methods

### Materials

P210 (KTTKQSFDLSVKAQYKKNKH), p210-PADRE (AKFVAAWTLKAAAKTTKQSFDLSVKAQYKKNKH), and PADRE (AKFVAAWTLKAAA) peptides were obtained from TAG Copenhagen. Alum (Pierce) was used as adjuvant and mixed together with p210-PADRE or PADRE peptide in 1:1 volume ratio. Peptide-alum mixture was freshly prepared prior to immunization.

Monoclonal mouse IgG2b against p210 was kindly provided by Abcentra (Los Angeles, CA) and monoclonal control InVivoPlus mouse IgG2b isotype control (Clone: MPC-11) was purchased from BioXCell.

### Animals

Female *Apoe*^*-/-*^ mice (B6.129P2-Apoe^tm1Unc^/J) were purchased from the Jackson Laboratory. Mice were immunized subcutaneously with 25 µg of p210-PADRE or equal molar amounts of PADRE at 15 weeks of age, followed by two booster injections after two and five weeks. Mice were fed a high-fat diet (HFD) (21% cocoa fat, 0.15% cholesterol from Lantmännen, Sweden), starting one week after the last booster injection, and sacrificed at twenty-seven weeks of age. In the passive immunization experiment, female *Apoe*^*-/-*^ mice were put on HFD at twelve weeks of age, and one week later administered with 500 µg IgG2b against p210 or isotype control intraperitoneally once a week for eight weeks before sacrifice. At the end of the study the mice were anaesthetized by inhalation of isoflurane (Isoba, Shering-Plough Animal health), killed by a 300 μL intraperitoneal injection of a solution consisting of 5 ml 0.9% NaCl, 1.1 ml 50 mg/ml ketamine (Ketaminol, Intervet, Stockholm, Sweden) and 0.9 ml 20 mg/ml xylazine (Rompun, Bayer, Leverkusen, Germany) followed by exsanguination by cardiac puncture. Aortas were perfused with PBS, pH 7.4 (Sigma, St Louis, MO, USA) before the aorta or the aortic arch were dissected free from connective tissue and mounted *en face* on ovalbumin (Sigma-Aldrich)-coated slides as described previously^31^ and stored in Histochoice (Amresco, USA) at 4 °C until further processing. Hearts were snapfrozen in liquid nitrogen and stored at − 80 °C before further processing. The studies were approved by the Malmö/Lund Animal Care and Use Committee, and all animal experiments were performed in accordance with relevant guidelines and regulations.

### Antibody analyses

Analyses of induced antibodies in mice were performed mainly as previously described by Vallejo et al^32^. p210, p210-PADRE, or PADRE peptides were used for coating (20 μg/mL of each in PBS) microtiter plates (Nunc MaxiSorp, Nunc) over night at 4 °C. Coated plates were washed with PBS with 0.05% Tween-20 and thereafter blocked with SuperBlock™ in Tris-buffered saline (Pierce) during 30 min at room temperature followed by an incubation of mouse plasma diluted 1:1000 in PBS-0.05% Tween-20 for 2 h at RT. After rinsing, bound antibodies were detected using alkaline phosphatase–conjugated goat anti-mouse IgG1 (BD Biosciences), IgG2c antibodies (Southern Biotech), or IgM antibodies (Vector Laboratories), which were incubated for 2 h at room temperature, and a color reaction was developed using phosphatase substrate kit (Pierce). The absorbance (405 nm) was measured and background absorbance was subtracted. Specificity assay of apoB100 p210-induced antibodies were performed by a competition ELISA. p210 peptide were coated in microtiter wells and binding of antibodies in plasma from p210-PADRE- or PADRE-immunized mice (dilution 1:1000) were competed by increasing concentrations of p210 peptide in solution (0, 1, 10 or 100 µg/ml). Bound antibodies were detected as described above.

### Lipid analysis

Plasma quantity of cholesterol and triglycerides were determined with Infinity Total Cholesterol and Triglyceride reagents from Thermo Scientific. Plasma oxidized low density lipoprotein (oxLDL) were determined using oxLDL ELISA Kit (SEA527Mu, Cloud-clone) according to the manufacturer.

### Immunohistochemistry and histochemistry

Frozen hearts were embedded in Tissue Tek (Sakura Fine Tek., Japan) and cross sections from the aortic root were collected with a thickness of 8 µm. Plaque area were analyzed in Oil red O stained *en face* preparations of the aorta or the aortic arch as described previously^12^. Subvalvular plaques were stained for macrophages (anti-CD68, 0.125 µg/ml, Abcam ab125212). Sections were permeabilized in 0.5% Triton X-100 (Merck Millipore) in PBS, and endogenous peroxidases were quenched by incubation in 3% H_2_O_2_ in PBS. Sections were blocked with serum in PBS followed by incubation with the primary antibody overnight at 4ºC in a pre-wet chamber. For detection, secondary biotinylated antibodies were applied for 1 h at room temperature, and bound antibodies were detected by VECTASTAIN Elite ABC HRP Kit (Vector Laboratories) followed by ImmPACT DAB Peroxidase (HRP) Substrate kit (Vector Laboratories). Sections were counterstained using Mayer´s hematoxylin (Histolab Products AB, Sweden). Aldehyde-modified (MDA)-LDL staining in subvalvular plaques was performed by incubating sections with 1% H_2_O_2_, followed by blocking with 10% bovine serum albumin and biotin-avidin (Vector Laboratories), before addition of biotinylated anti-MDA-LDL, 10 µg/ml (Biotechne). Bound antibodies were detected as described above. Collagen content was determined by van Gieson staining (van Gieson solution, HT254, Sigma-Aldrich). Subvalvular plaque area was determined in hematoxylin-stained cross-sections. Stained sections were scanned using an Aperio ScanScope digital slide scanner (Scanscope Console v8.2.0.1263, AmperioTechnologies, Inc., USA) and stained area was quantified blindly using Biopix Q (Biopix AB) software, and presented as percentage stained area of total plaque area or total vessel area.

### Splenocyte isolation and stimulation

Single-cell suspensions of splenocytes were prepared by pressing spleens through a 70-μm cell strainer (BD Falcon) as previously described^32^. Erythrocytes were removed using red blood cell lysing buffer (Sigma-Aldrich). Cells were cultured in culture medium containing 10% heat-inactivated fetal calf serum, 1 mmol/L sodium pyruvate, 10 mmol/L Hepes, 50 U of penicillin, 50 µg/mL streptomycin, 0.05 mmol/L β-mercaptoethanol, 2 mmol/L L-glutamine (RPMI 1640, GIBCO) and CD3/CD28 activating beads (Life Technologies, 11452D), 1 × 10^6^ cells/well, in 48-well plates (Corning) for 24 h.

### Cytokine analysis

Cytokines in plasma or from CD3/CD28 stimulated splenocytes were measured using Luminex multiplex technology (Millipore) according to the manufacturer.

### LDL isolation and labeling

LDL was isolated from human plasma by density gradient ultracentrifugation^33^. The plasma came from healthy volunteers not involved in the study (Sahlgrenska university hospital blood bank). Isolated LDL was filtered (0.2 μm) and dialyzed for 48 h at 4 °C in PBS pH 7.4 with 1/20 volumes of 0.2 M sodium bicarbonate pH 9.0, and then labeled with Atto594-fluorochrome according to manufacturer’s instructions (AD 594-31, Atto-tec, Siegen, Germany) and dialyzed in 50 mmol/L Tris, 40 mmol/L NaCl, pH 7.0 as previously described^34^. Until use, Atto594-LDL was stored at 4 °C protected from light.

### Ex vivo* LDL-binding experiments*

Intimal hyperplasia was induced in the right common carotid artery of C57Bl/6 wild-type mice (n = 4) as previously described^35^. After 3 weeks, the carotid arteries were harvested, mounted in OCT-medium, and frozen in liquid nitrogen. 10 μm cryo-sections were cut, air-dried for 1 h and stored in -20 °C until use. For LDL binding experiments, Atto594-labeled human LDL (1 mg/ml ApoB100) was pre-incubated with monoclonal p210 antibody (0.87 mg/ml), isotype control antibody (0.87 mg/ml), or control buffer solution only (38 mmol/L Tris, 47 mmol/L NaCl, 0.65 mmol/L KCl, 2.4 mmol/L phosphate buffer, 5% glycerol, pH 7.0) for 30 min at 37 °C. Meanwhile, tissue sections were pre-incubated for 30 min with either PBS or Site B mimicking peptide (700 μM, sequence: RLMRKRGLKLATAVSLTNK, Caslo, Denmark) dissolved in PBS. The sections were then washed in sample buffer (50 mmol/L Tris, 40 mmol/L NaCl, pH 7.0) and incubated with the antibody-treated LDL for 45 min at 37 °C. Sections were then washed in sample buffer, fixed in 2% paraformaldehyde in sample buffer for 5 min, washed twice in sample buffer, and mounted with ProLong Gold antifade reagent. Images were acquired with Carl Zeiss AxioImager.Z2 microscope with plan‐apochromat 40x/1.4 oil objective (Carl Zeiss Microscopy, Germany). Visiopharm image analyzing software was used for image analysis (Visiopharm, Hoersholm, Denmark). The mean fluorescence intensity (Atto594 signal) of the intimal vessel wall layer (intimal hyperplasia) was acquired and background fluorescence (tissue sections without LDL-594 incubation) was deducted.

### Binding of p210 mAb to native and MDA-modified p210, apoB100, and LDL

Binding of p210 mAb to p210, MDA-p210, p265, and MDA-p265 was measured using ELISA. Briefly, peptides p210, MDA-p210, p265, and MDA-p265 were coated at 2 µg/ml on Greiner HB plates, blocked with 0.5% polyvinyl alcohol in PBS, and p210 mAb was added at concentrations ranging from 1.37 to 1000 ng/ml. Bound antibodies were detected using horse radish peroxidase conjugated goat anti-mouse IgG (Southern Biotechnologies, 1090–05) followed by TMB substrate (Moss Substrates). Reaction was stopped by addition of 0.5 N HCl, and absorbance was read at 450 nm. The binding of p210 mAb to native and MDA-LDL was determined using competitive ELISA where p210 was coated at a concentration of 1 µg/ml and binding of p210 mAb (12.5 ng/ml) was competed by addition of native or MDA-LDL (Academy Bio-Medical Company, 20P-MD-L102) at concentrations 1.56–100 µg/ml. Bound antibodies were detected by as described above. In addition, binding of mAb p210 to native and MDA-apoB100 was measured on real time basis using OctetRed96 label-free binding assay. Bio layer interferometry (BLI), the underlying technique for OctetRed96, measures changes in an interference pattern generated from visible light reflected from an optical layer and a biolayer containing proteins of interest. The interference is measured in terms of nm shift and is proportional to extent of interaction or amount bound. Briefly, the binding assays were performed as follows: The mAb p210 was captured using pre-conditioned anti-mouse IgG Fc Capture (AMC) antibody coated dip and read disposable biosensors. Sensors were then dipped into baseline buffer (assay buffer), then into the wells containing native or MDA-apoB100 (10 nM), for the association to occur and then moved to assay buffer (PBS, 0.05% Tween-20), for dissociation to occur. The association and dissociation of the analyte were observed on a real time basis. From the observed rate constants, equilibrium constant (K_D_) was calculated as k_d_/k_a_ with 1:1 global fitting analysis using the data analysis software version 9.0.

### Statistical analysis

Statistical analyses were performed using Graphpad Prism (GraphPad Software, USA). Data are presented as individual mice, together with mean ± standard deviation (SD) for normally distributed variables or together with median (interquartile range) (IQR) for non-normally distributed variables. Differences between groups were analyzed using unpaired two-tailed t-test or with Mann–Whitney test depending on the distribution of the variable as indicated in the figures legends. *p* < 0.05 was considered as statistically significant.

## Supplementary Information

Supplementary Information
