# Supplementary material for: Antibodies against apoB100 peptide 210 inhibit atherosclerosis in apoE-/- mice
Source: Sci Rep. 2021 Apr 27;11:9022. doi: 10.1038/s41598-021-88430-1 (PMC8079692; doi:10.1038/s41598-021-88430-1)
Supplement: Supplementary file 1 — Supplementary Information [file 41598_2021_88430_MOESM1_ESM.pdf]

Supplementary information

Antibodies against apoB100 peptide 210 inhibit atherosclerosis in apoE$^{-/-}$ mice

Pontus Dunér[1], Ingrid Yao Mattisson[1,2], Per Fogelstrand[3], Lars Glise[3], Stacey Ruiz[4], Christopher Farina[4], Jan Borén[3], Jan Nilsson[1], Eva Bengtsson[1]

[1]Department of Clinical Sciences Malmö, Clinical Research Centre, Lund University, Sweden

[2]Current address: Redoxis AB, Medicon Village, Lund, Sweden

[3]Department of Molecular and Clinical Medicine, Wallenberg Laboratory, Institute of Medicine, the Sahlgrenska Academy at University of Gothenburg and Sahlgrenska University Hospital, Sweden

[4]Abcentra LLC, Los Angeles, CA, USA

Supplementary Table 1

Plasma lipids and body weight in PADRE and p210-PADRE immunized mice

	**PADRE**	**P210-PADRE**
Cholesterol (mg/dl)	813±109	744±85
Triglycerides (mg/dl)	100±17	95±39
Oxidized LDL (µg/ml)	513±84	672±190
Body weight (g)	29±5	28±7

Data are shown as mean±standard deviation.
There were no significant differences between the groups.

Supplementary Table 2

Plasma lipids and body weight in mice immunized with p210 murine monoclonal antibody or control antibody

	Ctrl mAb	P210 mAb
Cholesterol (mg/dl)	540±103	490±44
Triglycerides (mg/dl)	50±7.7	52±8.5
Oxidized LDL (ng/ml)	27 (23-25)	40 (31-54)
Body weight (g)	23 (23-25)	25 (23-28)

Data are shown as mean±standard deviation or median (IQR).
There were no significant differences between the groups.

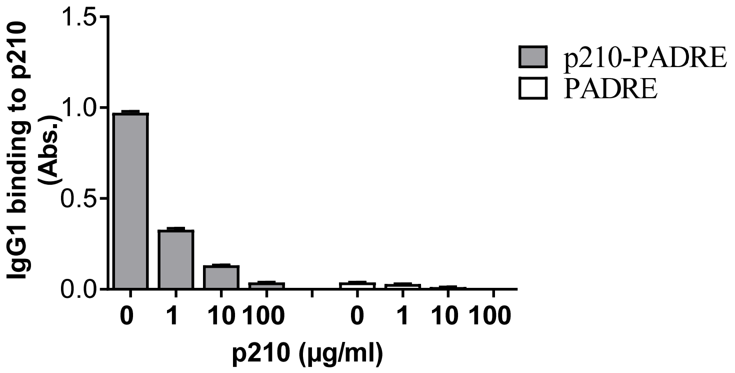

Supplementary figure 1.
ApoE$^{-/-}$ mice immunized with native p210-PADRE develop p210-specific IgG1 antibodies.
Pooled plasma (dilution 1:1000) from p210-PADRE or PADRE immunized mice were incubated with different concentrations of p210 prior binding to p210 coated microtiter wells. Data are shown as means±SD.

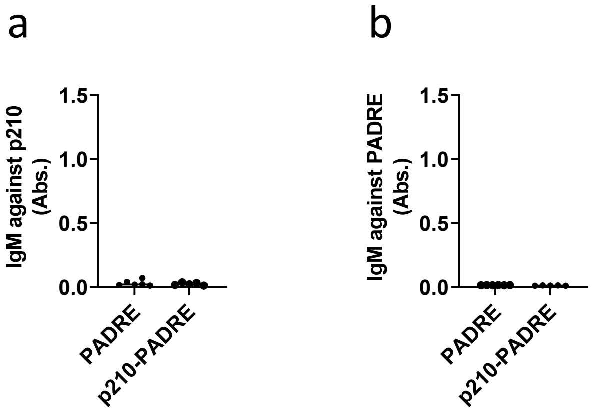

Supplementary figure 2.

ApoE$^{-/-}$ mice immunized with native p210-PADRE do not develop IgM antibodies.

ApoE$^{-/-}$ mice were immunized with native p210-PADRE or PADRE peptide. IgM antibodies against p210 (a), or PADRE (b) (plasma dilution 1:1000) from immunized mice were analyzed in ELISA. Data are depicted as individual mice.

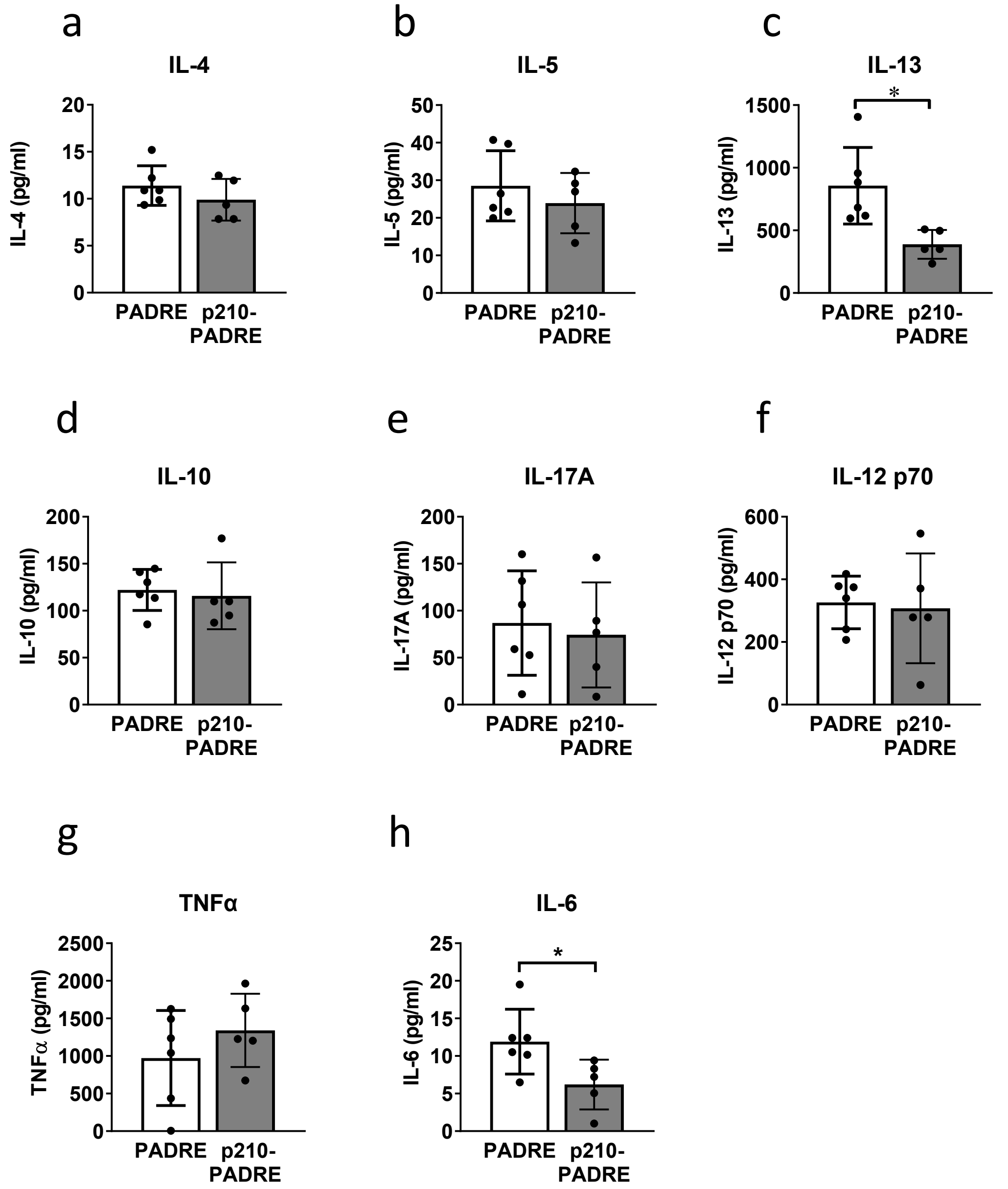

Supplementary figure 3.

Systemic cytokine levels of mice immunized with native p210-PADRE or PADRE. ApoE$^{-/-}$ mice were immunized with native p210-PADRE or PADRE peptide, and Th2, Treg, Th17, Th1, and inflammatory cytokines were analyzed in plasma. Data are depicted as individual mice, with bars indicating mean±SD (a-h). Unpaired t-test. *p<0.05.

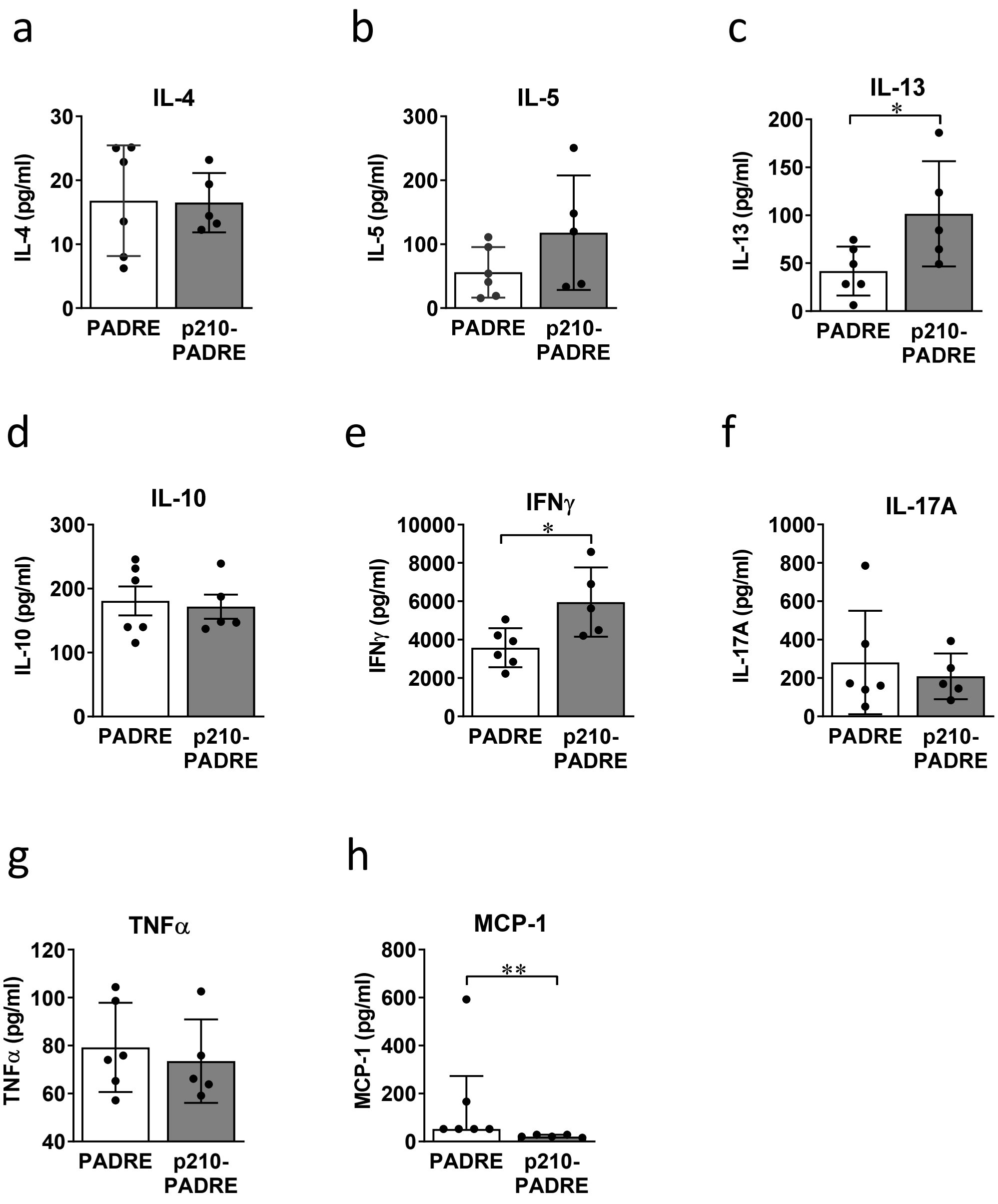

Supplementary figure 4.

Cytokine secretion from splenocytes of mice immunized with native p210-PADRE or PADRE.

$ApoE^{-/-}$ mice were immunized with native p210-PADRE or PADRE peptide, and Th2, Treg, Th1, Th17, and inflammatory cytokines were analyzed in medium from CD3/CD28 stimulated splenocytes. Data are depicted as individual mice, with bars indicating mean±SD (a-g) or median (IQR) (h). Unpaired t-test or Mann-Whitney test. *p<0.05, **p<0.01

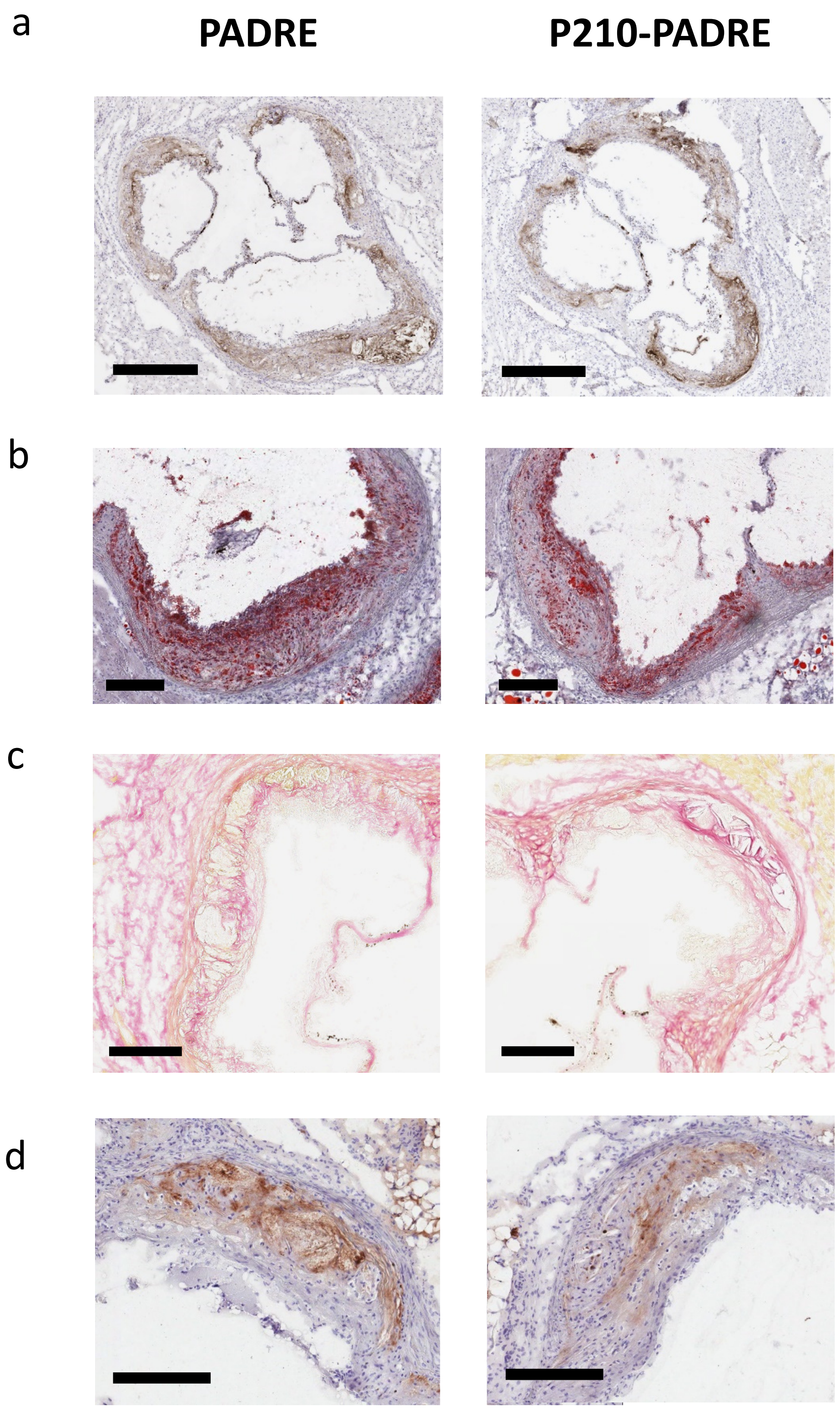

Supplementary figure 5.
Subvalvular plaques from ApoE$^{-/-}$ mice immunized with p210-PADRE or PADRE. Representative images of macrophage (CD68) (a), lipid (Oilred O) (b), collagen (van Gieson) (c), and MDA-LDL (anti-MDA-LDL) (d) staining. Scale bars 500 µm (a), 200 µm (b-d).

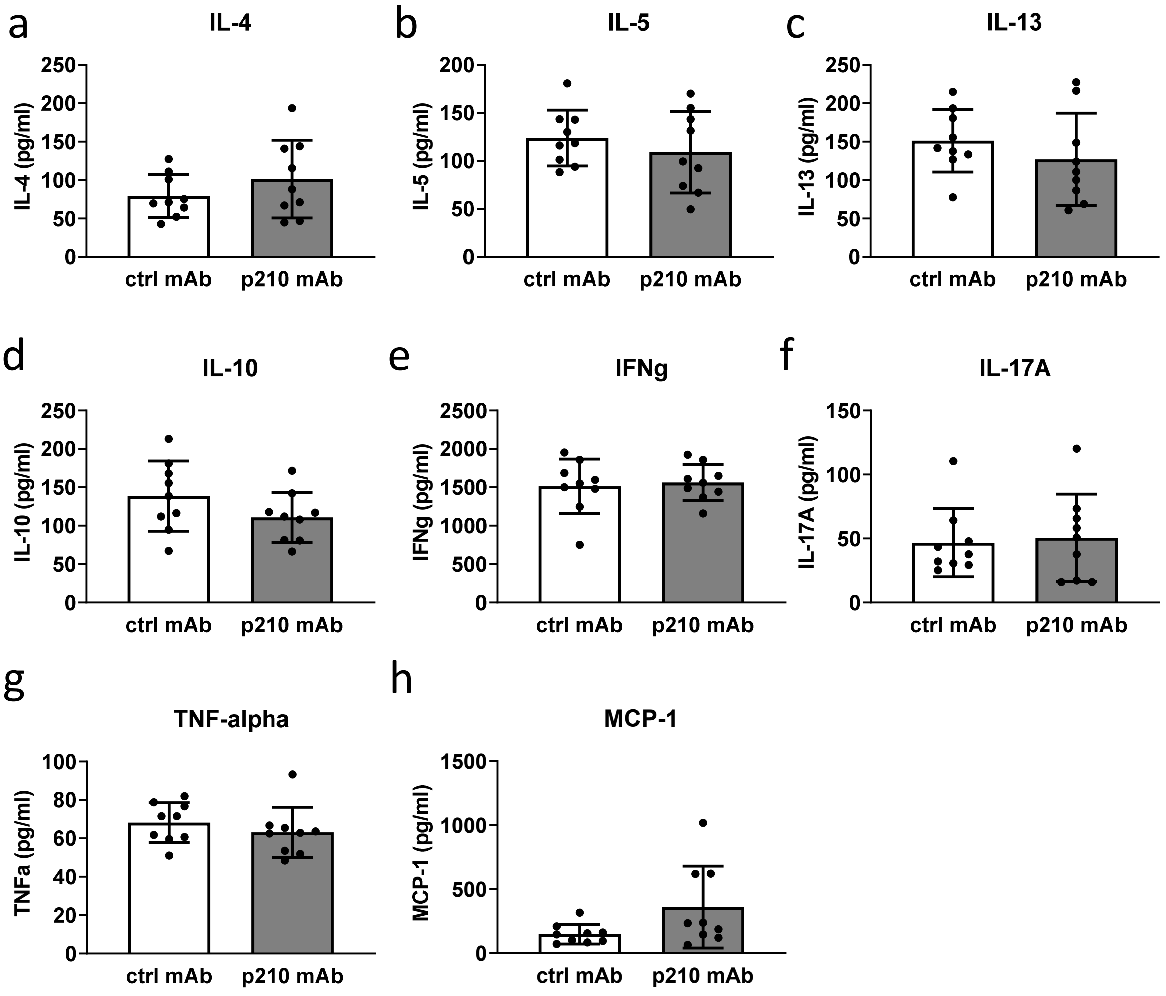

Supplementary figure 6.
Immunization with IgG against native p210 do not affect cytokine secretion of splenocytes. ApoE$^{-/-}$ mice were immunized with monoclonal IgG against native p210 or control IgG. Th2, Treg, Th1, Th17, and inflammatory cytokines were analyzed in medium from CD3/CD28 stimulated splenocytes. Data are depicted as individual mice, with bars indicating mean±SD (a-e) or median (IQR) (f-h).

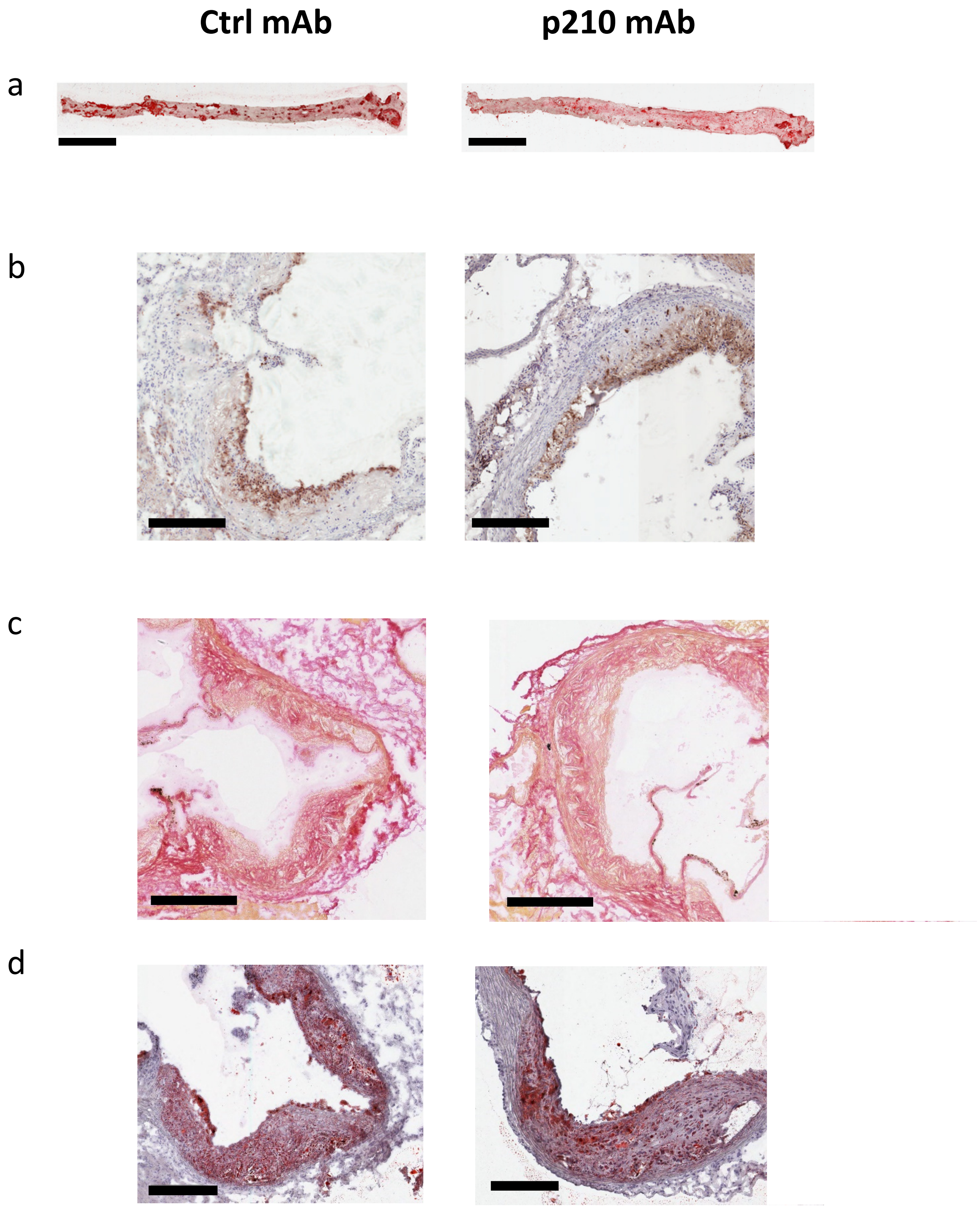

Supplementary figure 7.

Aortic and subvalvular plaques from ApoE$^{-/-}$ mice immunized with p210 mAb or ctrl mAb. Representative images of *en face* preparations of the aorta stained with Oilred O (a), and subvalvular plaques stained for macrophages (CD68) (b), collagen (van Gieson) (c), and lipids (Oilred O) (d). Scale bars 5 mm (a), 200 µm (b,d) or 300 µm (c).
